# Incidental detection of two adult gravid filarial worms in breast: a case report

**DOI:** 10.1186/s13104-017-2709-3

**Published:** 2017-08-16

**Authors:** Santosh Tummidi, Kanchan Kothari, Roshni Patil, Shruti S. Singhal, Pooja Keshan

**Affiliations:** Department of Pathology, Seth GSMC & KEMH, Parel, Mumbai, Maharashtra 4900012 India

**Keywords:** Adult gravid worm, Embryoid forms, Filaria, Breast, Fine needle aspiration

## Abstract

**Background:**

Microfilaria is a major public health problem in tropical and subtropical countries and is an endemic problem in India. *Wuchereria bancrofti* is the commonest filarial infection. In some lesions, microfilariae and adult filarial worm have been incidentally detected in fine-needle aspirates.

**Case presentation:**

A 35 year old hindu female presented with lump in upper outer quadrant of left breast. Fine needle aspiration revealed two adult gravid female filarial worms.

**Conclusion:**

To our knowledge this is the first ever case report to demonstrate two live gravid female and embryoid forms in wet mount preparation.

**Electronic supplementary material:**

The online version of this article (doi:10.1186/s13104-017-2709-3) contains supplementary material, which is available to authorized users.

## Background

Filariasis has been a disabling parasitic disease worldwide, particularly in tropical and sub-tropical countries of the world. The disease is fairly endemic in India. The commonest causative agents are two closely related nematodes, *Wuchereria bancrofti* and *Brugia malayi* accounting for 98% of cases. Human beings serve as definitive host for the parasite while culex mosquitoes serve as the intermediate host [1, 2]. It has predilection for lower limbs, spermatic cord and epididymis while breast, thyroid, body fluids and skin are unusual sites [3–5]. Despite the fact that large numbers of people are at risk and wide varieties of tissues are affected, adult worm is rarely found in fine-needle aspiration cytology (FNAC) smears. Till date there are only 4 cases demonstrating adult female worm in breast lump aspirates.

## Case presentation

**Fig. 1:**
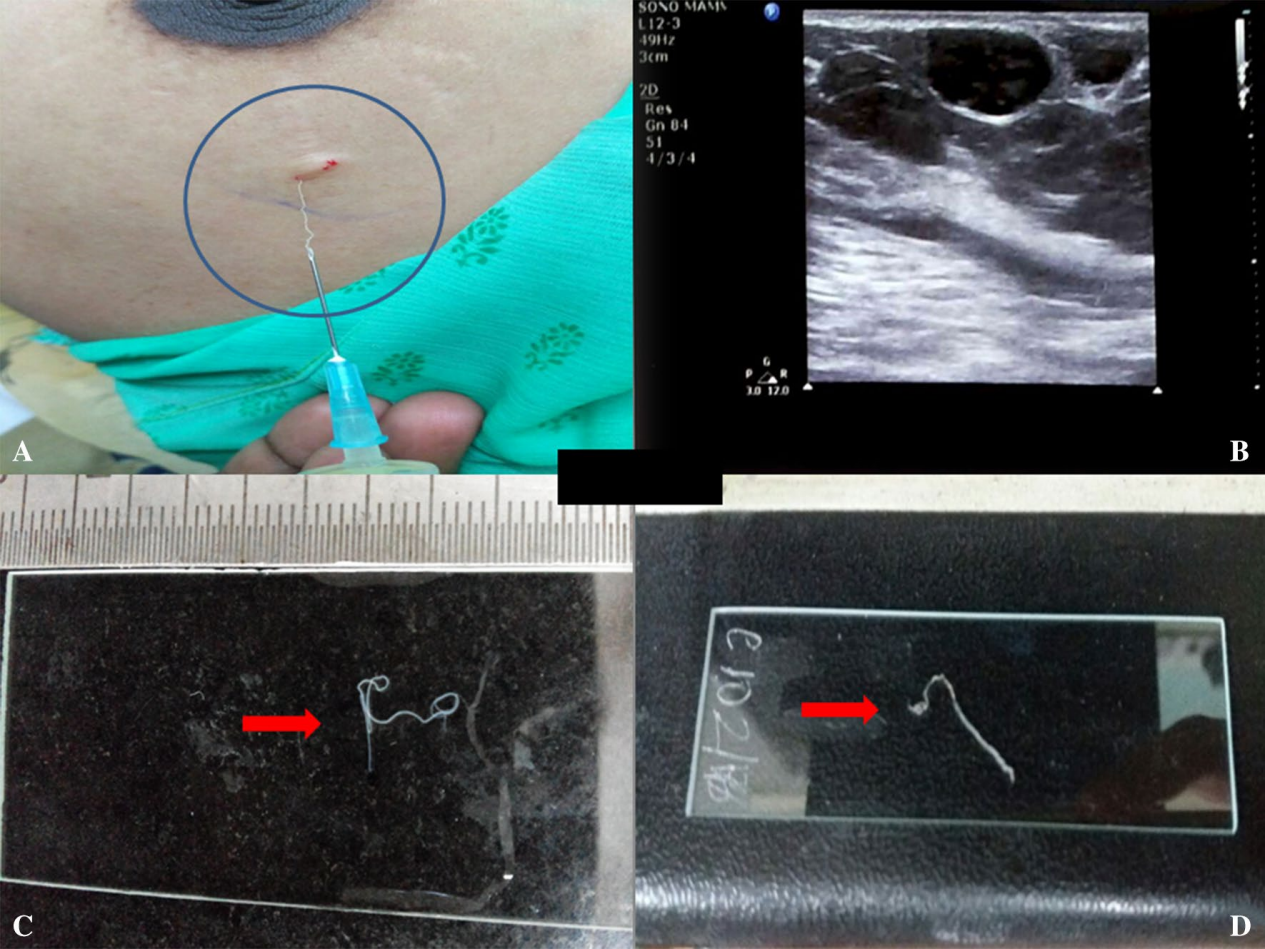
**A** Aspiration of the parasite from lump in the upper outer quadrant of left breast; **B** sonomammography with cystic lesion measuring 1 × 0.73 cm and filarial dance sign; **C** adult filarial female worm on slide after 1st attempt of fine needle aspiration; **D** another adult filaria worm on 2nd attempt of fine needle aspiration

A 35 year old hindu female came with complaints of lump in the left breast since 3 years with pain since 4 days. On examination, a lump was present in upper outer quadrant of left breast measuring 1 × 1 cm (Fig. 1A). There was no axillary lymphadenopathy and contralateral breast was normal. She had no history of fever/trauma/nipple discharge/variation in size of lump. All hematological parameters were normal except for mild anemia. Bilateral sonomammography revealed cystic lesion measuring 1 × 0.7 cm containing multiple curvilinear echoes, with “filarial dance” in the subcutaneous plane of left breast, at around 2 o’clock position (Fig. 1B). She was then referred for cytology diagnosis of the lump.

**Fig. 2:**
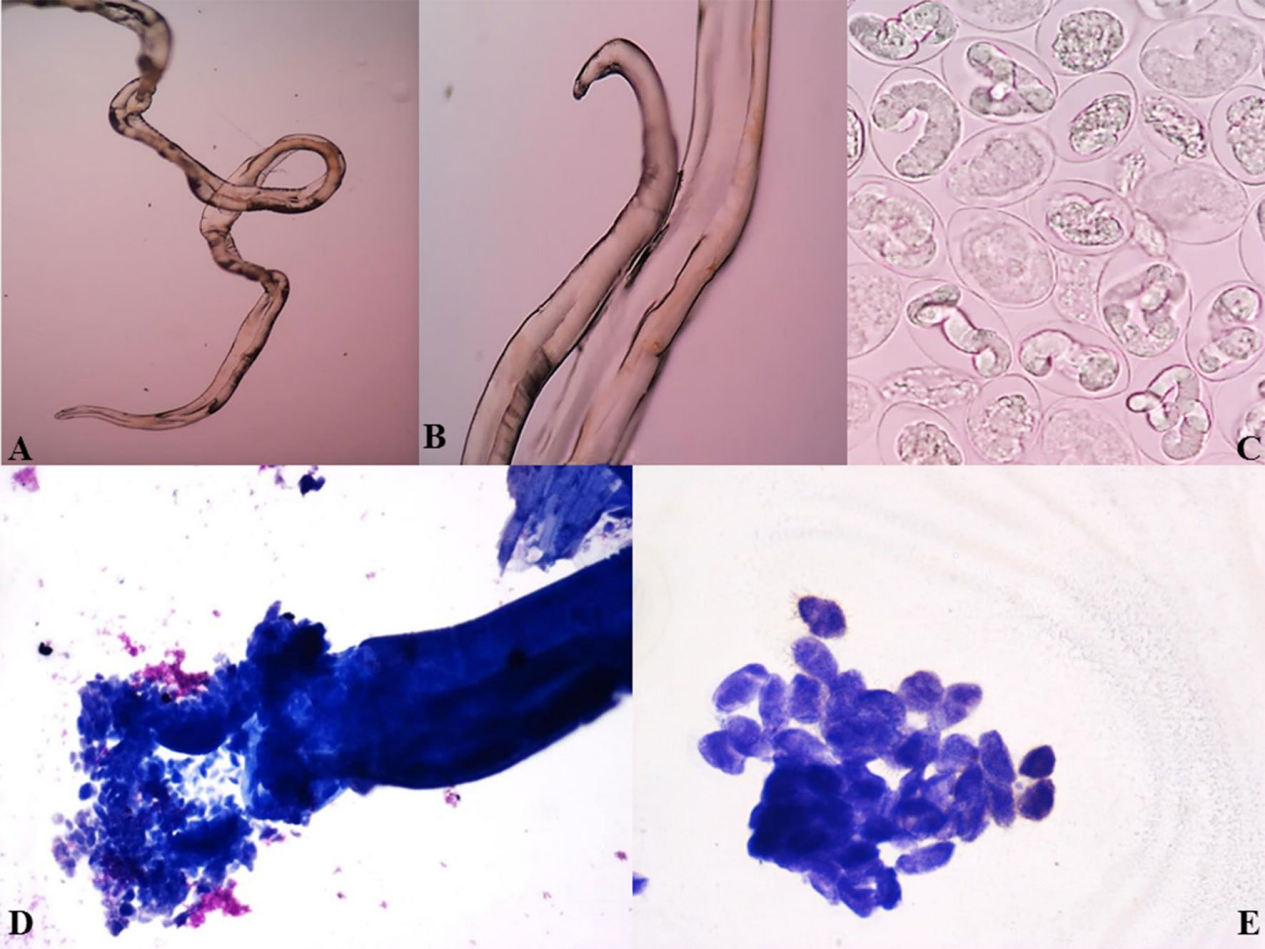
**A** Wet mount showing one end of adult female worm; **B** wet mount of female worm other end intact; **C** wet mount after nick in intestine showed many embryoid forms of parasite coming out in thousands; **D** toluidine blue stain revealed adult gravid female worm intestine with embryoid forms coming out. **E** Embryoid forms in clusters (Tol Blue, ×40)

**Fig. 3:**
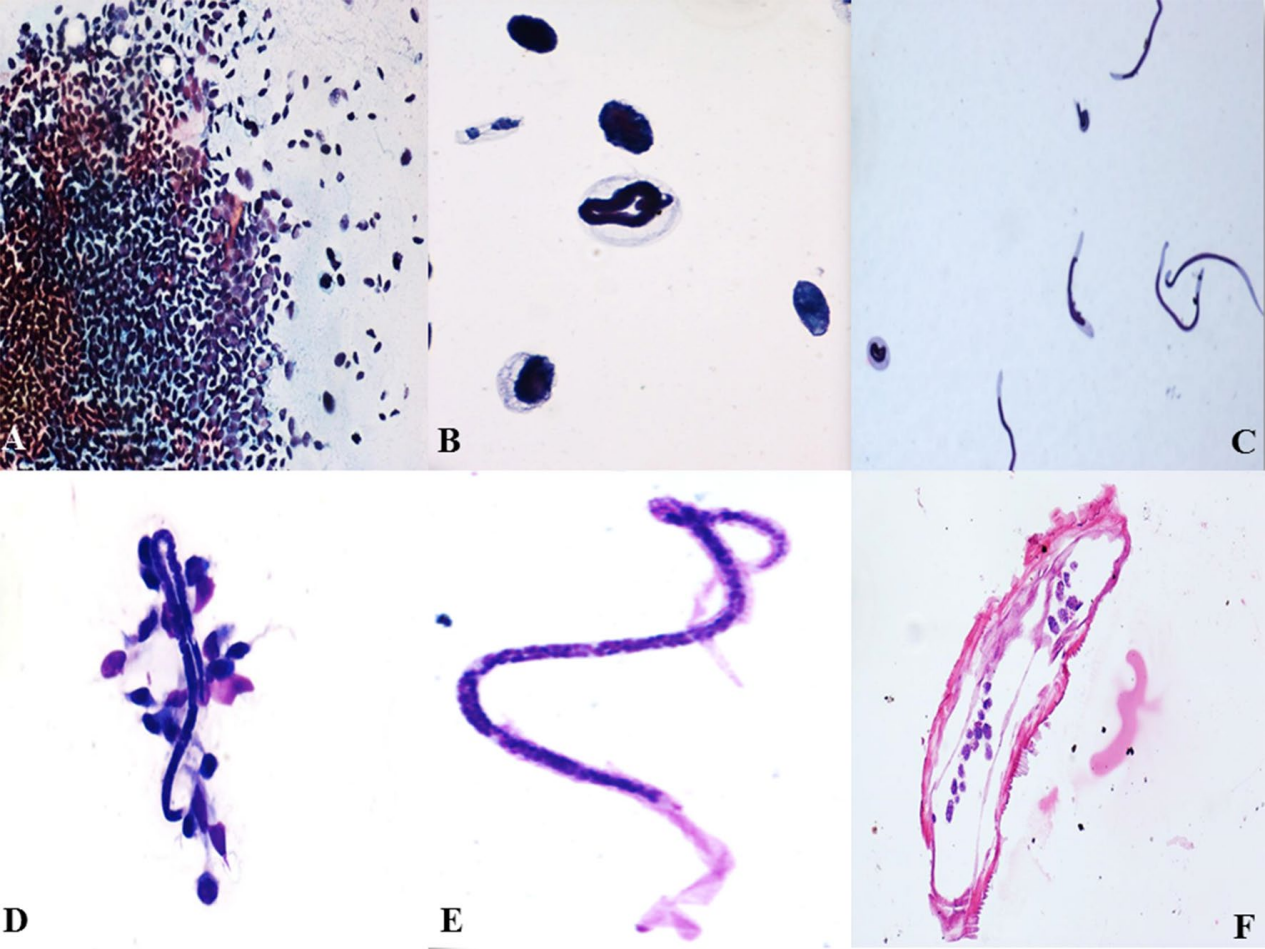
**A** Microscopy showed many embryoid forms (PAP, ×10); **B** many coiled microfilariae and embryoid forms seen (PAP, ×40); **C** uncoiled sheathed microfilariae with tail end being free of nuclei. **D** Granulomatous reaction to filarial worm along with few lymphocytes, polymorphs and histiocytes along with a coiled microfilariae (Geimsa, ×10); **E** microfilaria with sheath and tail free of nuclei (Geimsa, ×40); **F** cell block preparation showing adult worm with many embryoid forms within intestinal cavity (H&E, ×40)

FNA from the lump was done using 23 gz needles. During the procedure, we were able to aspirate two adult filarial gravid female worms along with 2 ml of granular turbid material. The worms were put on a plain slide and measured to be 3 and 4.5 cm respectively (Fig. 1C, D). Wet mount preparation of the parasite was done using normal saline. Microscopy showed two adult female worms, one with intact head and tail ends while other was incomplete. The worm was then given a nick in intestinal region from where embryoid forms of the parasite were seen to release out in thousands (Fig. 2A–C) (Additional file 1: Video 1). Smears were then stained with Toluidine blue, Papanicoloau and Geimsa. Microscopy revealed adult gravid female worm with many embryoid forms as well as coiled and uncoiled sheathed microfilariae with tail end being free of nuclei. A granulomatous reaction along with few lymphocytes, polymorphs and histiocytes was also seen (Figs. 2D, E, 3A–E). Cytological diagnosis was given as breast filariasis due to *W. bancrofti*. One of the adult worms was also processed for histopathology and revealed similar features (Fig. 3F).

## Discussion

The medical literature documents filariasis back to 600 BC when Sustruta recognized the clinical manifestation of elephantiasis and referred it as *elephantiasis arabicum* [3]. These nematodes belong to the order Spirurida and superfamily Filarioidia [1]. Adult worms are found in lymphatic vessels of lower limbs, spermatic cord, epididymis, testis, retroperitoneum and breast of humans, while the larval forms (microfilaria) may circulate in the peripheral blood [4]. The adult female worms are usually of 80–100 mm length, ovoviviparous giving birth to ova containing microfilariae whereas males are 40 mm in length [1].

The clinical spectrum of lymphatic filariasis ranges from only peripheral blood eosinophilia to lymphangitis finally terminating in elephantiasis. The parasite is usually found in lymphatics sometimes causing obstruction with cystic change. The probable explanation for the mechanism of microfilariae reaching extravascular tissue spaces from microcirculation is by crossing the vessel wall by their boring ability [5].

Although there are reports of accidental detection of microfilaria using aspiration cytology in various sites like spermatic cord [6], epididymis [6], testis, lymph nodes [7], retroperitoneum, soft tissue, breast [3] and bone marrow etc. [5], reports of adult worms in cytological aspirates are sparse. Pandit et al. [8] and Azad et al. [9] reported the presence of adult filarial worms in soft tissue swellings. Satpathi et al. [10], Pal et al. [11], Chakarbarti et al. [3] and Naorem et al. [12] have reported cases of adult filarial worms in the breast aspirate. Our case is an addition to this list of rare occurrence of adult gravid female worm in breast. Also in the present case, the length of gravid female worm was 4.5 cm (Intact worm).

Adult female worms of the two above mentioned species cannot be distinguished though adult male worms show minor differences. Species diagnosis thereby is made on the basis of morphology of the microfilaria [1, 4]. The microfilaria of *W. bancrofti* is larger in size and possesses smooth body curve, its body nuclei are well-defined, discrete, round and uniform in size compared to that of *B. malayi*, which is smaller in size, possesses secondary kinks and blurred and intermingled nuclei. Tail tapers to a delicate point with absent terminal nucleus in *W. bancrofti* whereas later it is more bulbous and has two distinct terminal nuclei. However, differentiation between these two nematodes is not clinically important as the mode of treatment remains same [2, 4].

The methods of detection are demonstrating the microfilariae, adult worm, circulating filarial antigen (CFA) and ultrasound with or without color doppler demonstrating the filarial dance sign [2] We had radiological, cytological and histopathological investigations for correlation. Filariasis can be cured by administration of Diethylcarbamazine citrate (DEC) [2].

## Conclusion

Our case report is to increase awareness of filariasis in tropical countries such as India where it’s endemic. Filariasis should be considered as a differential diagnosis in subcutaneous lumps. Careful clinical, radiological and cytological correlation is very important in prompt recognition of the disease and institution of specific treatment.

## Additional file

**Additional file 1.** Microscopy showing the parasite with numerous embryoid forms comming out.
